# Development and characterization of positively selected brain-adapted SIV

**DOI:** 10.1186/1743-422X-2-44

**Published:** 2005-05-12

**Authors:** Peter J Gaskill, Debbie D Watry, Tricia H Burdo, Howard S Fox

**Affiliations:** 1 Department of Neuropharmacology, The Scripps Research Institute, 10550 N. Torrey Pines Road, La Jolla, CA, 92037, USA

## Abstract

HIV is found in the brains of most infected individuals but only 30% develop neurological disease. Both viral and host factors are thought to contribute to the motor and cognitive disorders resulting from HIV infection. Here, using the SIV/rhesus monkey system, we characterize the salient characteristics of the virus from the brain of animals with neuropathological disorders. Nine unique molecular clones of SIV were derived from virus released by microglia cultured from the brains of two macaques with SIV encephalitis. Sequence analysis revealed a remarkably high level of similarity between their *env *and *nef *genes as well as their 3' LTR. As this genotype was found in the brains of two separate animals, and it encoded a set of distinct amino acid changes from the infecting virus, it demonstrates the convergent evolution of the virus to a unique brain-adapted genotype. This genotype was distinct from other macrophage-tropic and neurovirulent strains of SIV. Functional characterization of virus derived from representative clones showed a robust *in vitro *infection of 174xCEM cells, primary macrophages and primary microglia. The infectious phenotype of this virus is distinct from that shown by other strains of SIV, potentially reflecting the method by which the virus successfully infiltrates and infects the CNS. Positive *in vivo *selection of a brain-adapted strain of SIV resulted in a near-homogeneous strain of virus with distinct properties that may give clues to the viral basis of neuroAIDS.

## Introduction

As the Acquired Immune Deficiency Syndrome (AIDS) pandemic continues to grow, the number of people affected by the neurological complications of human immunodeficiency virus (HIV) infection expands. Neurological complications, known collectively as neuroAIDS, affect approximately 30% of those infected with HIV [1]. Although our knowledge of the process by which HIV causes brain disease is constantly expanding, we still have only a limited understanding of the underlying pathogenic mechanism leading to disease in the central nervous system (CNS). It has been shown that an increase in the population of brain macrophages is a significant pathological correlate of neurological disease [2], and that most strains of HIV isolated from the brains of individuals with neurological disease are macrophage tropic and utilize the CCR5 co-receptor [3,4]. Macrophages and microglia, related cells of monocytic lineage, are the only cell types consistently infected in the brains of HIV-infected individuals [5]. Damage to neurons is thus indirect, resulting from effects of viral proteins or products of infected macrophages. The ability of HIV to infect macrophages and microglia *in vitro *is predictive of its neuroinvasiveness [6] and infected monocytes/macrophages are thought to carry HIV into the brain as per the Trojan horse hypothesis [7,8].

Simian immunodeficiency virus (SIV) is closely related to HIV [9,10] and SIV infection of macaques can generate a neuroAIDS-like syndrome that mirrors neuroAIDS in humans, demonstrating the neuropathological hallmarks of neuroAIDS found in HIV-infected humans along with cognitive, motor, and neurophysiological impairments [11-15]. The similarities between HIV- and SIV-induced neurological disease in humans and macaques, in light of the ethical and practical limitations of performing neurological research in humans, make the rhesus macaque an excellent model for the study of neuroAIDS.

There are a variety of strains and molecular clones of SIV that have been used to study aspects of AIDS pathogenesis, many of which are derived from the SIVmac251 strain [16]. Of the molecular clones, the most commonly used is SIVmac239, derived from the SIVmac251 strain by animal passage and tissue culture proviral DNA cloning [17]. SIVmac239 is highly pathogenic *in vivo *and displays a very high infectious capacity for T cells, but not macrophages, *in vitro *[17]. Unlike T-cell-tropic strains of HIV, which utilize the CXCR4 but not the CCR5 co-receptor, the T-cell tropism of SIVmac239 may be based on its inefficient use of the relatively low cell-surface CD4 density on rhesus macrophages, rather than co-receptor specificity [18]. Yet this may not fully explain SIVmac239's lack of productive macrophage infection, since many studies have found efficient entry, but post-reverse transcriptional blocks in the SIVmac239 life cycle in macrophages [19-21].

Other studies have examined the molecular aspects of virus recovered *ex vivo *from macrophages late in infection, revealing specific nucleotide/amino acid changes in viral genes and their products, which are associated with high levels of infection of macrophages *in vitro *[22-25]. In studying SIV cellular tropism, another commonly used clone is SIVmac316, isolated from proviral DNA in lung macrophages of a macaque that died rapidly after infection with SIVmac239 [24]. Tropism studies with this clone and others like it essentially examine viral revertants, examining changes in the viral sequence in the context of the backbone of SIVmac239 [26].

We have taken an independent approach to examine viral properties of SIV in the CNS. Using the SIVmac251 stock, we performed a serial passage of cell-associated virus isolated from the CNS of infected monkeys, followed by production of a cell-free stock of virus from *in vitro *infected microglia [27,28]. In this manner, we utilized a forward selection of neuroinvasive variants that exist in, or arose from, the SIVmac251 stock. In this study we discuss the development and analysis of SIV clones derived from virus released by cultured microglia that were isolated from the brains of monkeys infected with microglia-passaged viral stock. Sequencing and characterization of viral tropism and infectious phenotype were then undertaken to analyze genomic and functional characteristics common to these brain-derived viruses.

## Results

### Molecular Cloning of Microglia-Derived SIV

A total of 43 clones of the 3' region of SIV were isolated from viral RNA found in the supernatant of microglia cultures derived from the brains of SIVmac182-infected macaques 225 and 321. Of these clones, 24 clones were from animal 225, and 19 clones from animal 321. A portion of gp41 was sequenced in each clone to insure the identity of the clones and to determine if any of the clones contained premature truncations due to stop codons in the gp41 region, a common finding in macrophage-tropic SIVmac239-derived clones. Sequence analysis confirmed that all of the clones were SIV, and that none had truncations in the gp41 region.

### Infectivity and Cytopathogenicity

Each of these 43 clones containing the 3' region of SIV was ligated to the 5' region of SIVmac239, and transfected into 174xCEM cells, a common indicator cell line for SIV infection. Cultures were observed daily for syncytia formation and monitored for infectious virus formation by p27Gag analysis of culture supernatants. Of the 43 viruses, 19 (13 from macaque 225 and 6 from macaque 321) induced syncytia formation in the cultures and/or tested positive for p27Gag production in the culture supernatant.

*In vitro *parameters of cytopathogenicity were then tested, using cells transfected with SIVmac239 as a positive control. SIVmac239 led to a very robust infection in 174xCEM cells, rapidly producing high levels of p27Gag (1.5 ng/ml) and syncytia. Pronounced cytopathic effects ensued, and the cells in the SIVmac239-transfected cultures were all dead by day 11 post-transfection. The microglia-derived molecular clones could be divided into three groups. A first group of five clones: 109, 129, 141, 142, and 169, produced the most consistent and robust infections, with all clones in this group generating syncytia, consistently high levels of p27Gag (above 1.5 ng/ml) and high levels of cell death by day 15 (Table 2).

**Table 2:** *In vitro *syncytia formation and viral antigen production. The molecular clones, derived from microglia of the indicated monkey, were tested by transfection and subsequent growth in 174xCEM cells.

**Clone**	**Monkey**	**Longest time to Syncytia Formation**	**Longest time to Detectable p27**
*Group 1*			
109	225	7 days	7 days
129	225	7 days	7 days
141	225	7 days	7 days
142	225	7 days	7 days
169	321	7 days	7 days
			
*Group 2*			
108	225	12 days	12 days
122	225	12 days	12 days
144	225	12 days	12 days
146	225	15 days	18 days
153	321	9 days	10 days
159	321	12 days	12 days
			
*Group 3*			
104	225	18 days	14 days
115	225	18 days	14 days
116	225	18 days	18 days
134	225	18 days	18 days
143	225	Never	18 days
164	321	12 days	12 days
171	321	Never	15 days
173	321	Never	15 days
			
*Control*			
SIVmac239		4 days	7 days

A second group of six clones: 108, 122, 144, 146, 153 and 159, also produced high levels of p27Gag and syncytia, although syncytia formation was slower than syncytia formation by the first group and these clones did not induce large amounts of cell death, with cells just beginning to die by 15–18 days post transfection. The remaining eight molecular clones that demonstrated signs of productive infection were 104, 115, 116, 134, 143, 164, 171 and 173. This group of clones 3 was considered least pathogenic of the three *in vitro *because they did not cause any detectable cell death and were unable to consistently generate syncytia and detectable p27Gag levels by day 18 experiments, although all of them did generate syncytia and high levels of p27Gag in at least one experiment, with the exception of 171 and 173, which did not generate syncytia despite p27Gag production.

### Sequence Analysis

The nine clones judged the most pathogenic *in vitro *were chosen for complete sequence analysis. These clones included all five from the most pathogenic group; 109, 129, 141, 142 and 169, as well as clones 108, 122, 153 and 159 from the second group. Clones from the second group were picked because they generated the highest levels of p27Gag in that group. Of the clones chosen, 108, 109, 122, 129, 141, and 142 were isolated from macaque 225 and clones 153, 159 and 169 were isolated from macaque 321. We fully sequenced the *env *and *nef *genes as well as the 3' LTR of each of these molecular clones. These sequences were used to develop a consensus sequence for all nine of the molecularly derived clones to be used for further analysis.

The nine clones showed a remarkable degree of similarity in the three gene products analyzed, with more differences in the TM portion of Env and in Nef than in the SU portion of Env. The nine clones differed from the consensus sequence by zero to six amino acids of 1,144 in amino acids in all three genes sequenced (Table 3). Comparison with other common molecular clones of SIV that were also derived from the SIVmac251 stock showed marked differences from the consensus sequence of the brain-adapted viruses in these regions (Table 3). The detail of these differences can be found in Table 4, showing that the brain-adapted genotype lacks the commonly seen truncation in gp41, and possesses 18 unique amino acids across Env and Nef.

**Table 3:** Comparison of encoded amino acids (AA) from clones described here (top) with other SIV molecular clones (bottom). The number (#) and percent (%) changes (Δ) in the indicated regions of Env and Nef are given.

**Clone**	**Monkey**	**Derived From**	**#AA Δ in gp120**	**#AA Δ in gp41**	**#AA Δ in Nef**	**Δ consensus in Env & Nef**
108	225	Viral RNA from Microglia supernatant	0	1	0	0.08%
109	225	Viral RNA from Microglia supernatant	1	0	0	0.08%
122	225	Viral RNA from Microglia supernatant	0	0	0	0.00%
129	225	Viral RNA from Microglia supernatant	1	1	0	0.17%
141	225	Viral RNA from Microglia supernatant	1	2	1	0.35%
142	225	Viral RNA from Microglia supernatant	1	0	1	0.17%
153	321	Viral RNA from Microglia supernatant	1	3	2	0.52%
159	321	Viral RNA from Microglia supernatant	0	2	2	0.35%
169	321	Viral RNA from Microglia supernatant	0	2	1	0.26%

SIVmac1A11	251-79	Proviral DNA from Tissue culture cells	28	8*	18	4.72%
SIVmac32H (pJ5)	32H	Proviral DNA from Tissue culture cells	24	14	19	4.98%
SIVmac316	316-85	Proviral DNA from Tissue culture cells	19	8*	N/A	3.07%**
SIV/17E-Fr	17E	Proviral DNA from Brain & Macrophages	22	11*	20	4.63%
SIVmac239	239-82	Proviral DNA from Tissue culture cells	18	14	15	4.11%

*truncated gp41, **Env only.

**Table 4:** Predicted amino acid residue at the indicated location in the SU region of Env. Bold indicates unique amino acids in clones 129 and 169.

**Env – gp120**	**67**	**79**	**127**	**132**	**134**	**135**	**144**	**153**	**176**	**178**	**309**	**382**	**385**	**475**	**511**
SIVmac239	V	N	I	S	T	S	M	A	K	D	M	G	D	G	D
SIVmac316	M	N	I	S	T	S	M	A	E	D	M	R	D	G	D
SIV17E-Cl	M	N	I	S	T	S	M	A	N	D	I	R	N	G	D
SIV17E/Fr	M	N	I	S	T	S	M	A	N	D	I	R	D	G	D
SIVmac32H	L	E	L	P	A	-	M	T	K	D	M	R	D	G	D
SIVmac1A11	L	E	S	A	P	-	M	V	K	D	I	G	D	G	N
Clone 129	L	**D**	**S**	S	T	**P**	**V**	**V**	K	**G**	I	R	D	**R**	N
Clone 169	L	**D**	**S**	S	T	**P**	**V**	**V**	K	**G**	I	R	D	**R**	N

*sequence not available, – no amino acid residue.

Additional sequence analysis was performed on the gp41 cytoplasmic tail regions of SIVmac251, SIVmac182 and cDNA derived from the supernatant of microglia from macaques 225 and 321. These reactions were performed on the cytoplasmic tail because of the variable sequence and frequent truncations found in this region and used a different set of primers than previous sequencing reactions in order to serve as independent confirmation of the observed amino acid changes. The brain-adapted viruses developed a unique sequence in this area, with 4 synonymous and 14 non-synonymous changes in the gp41 cytoplasmic tail regions of both 225 and 321 cDNA when compared with the original progenitor strain SIVmac251. There were also two synonymous and five non-synonymous changes found when comparing 225 and 321 cDNA with that of their immediate progenitor, SIVmac182. The synonymous and non-synonymous changes from both SIVmac251 and SIVmac182 were identical in uncloned PCR products from both 225 and 321 microglia supernatants, and the resulting amino acid changes in gp41 can be seen in Table 5.

**Table 5:** Predicted amino acid residue at the indicated location in the TM region of Env. Bold indicates unique amino acids in clones 129 and 169.

**Env – gp41**	**573**	**631**	**676**	**713**	**734**	**737**	**741**	**751**	**752**	**760**	**764**	**767**	**785**	**802**	**821**	**850**	**855**
SIVmac239	K	K	D	M	Q	I	P	R	D	S	W	E	S	L	T	G	T
SIVmac316	T	K	D	V	Q	I	P	G	D	S	W	Stop	-	-	-	-	-
SIV17E-Cl	K	K	N	V	Q	*	*	*	*	*	*	*	*	*	*	*	*
SIV17E/Fr	K	K	D	M	Q	I	P	G	D	S	Stop	-	-	-	-	-	-
SIVmac251	K	N	D	M	Q	I	P	G	D	S	W	E	S	L	T	G	T
SIVmac32H	K	D	D	M	Q	I	P	G	D	S	W	E	S	L	T	G	T
SIVmac1A11	K	K	D	M	Stop	-	-	-	-	-	-	-	-	-	-	-	-
Clone 129	K	D	D	M	Q	**T**	**Q**	G	**D**	**R**	W	E	**N**	**F**	**A**	**R**	**T**
Clone 169	K	D	D	M	Q	**T**	**Q**	G	**G**	**R**	W	E	**N**	**F**	**A**	**R**	**A**

*sequence not available, – no amino acid residue.

### Macrophage Infection

It has previously been shown that a majority of viruses isolated from the brains of individuals with neurological disease are macrophage tropic, and the ability to infect macrophages is thought to be key in the induction of neurological disease. Because the molecularly cloned viruses were all isolated from the brains of rhesus macaques that suffered from encephalitis, we hypothesized that these viruses were macrophage tropic. To test this hypothesis, we isolated macrophages from rhesus macaque PBMC and inoculated them with six of the molecularly cloned viruses. Three of the viruses that were fully sequenced, clones 108, 122 and 142, were dropped from this analysis because their sequences were greater than 99% similar to another clone being used for these infections. In order to generate more uniform results between experiments, all inoculations were performed using spinoculation. Spinoculation effectively eliminates potential differences in viral infection resulting from viral attachment to the cell, because it moves viruses directly onto their cellular targets [29,30].

**Figure 1:**
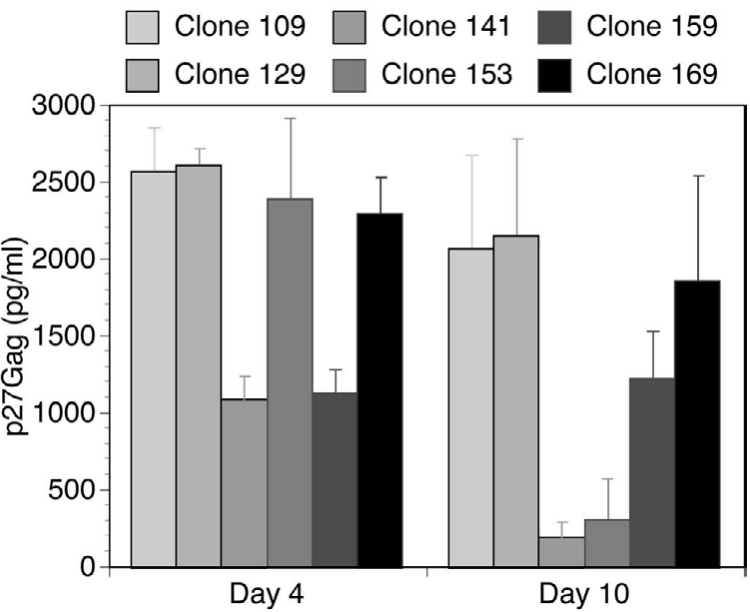
Viral replication in macrophages of six brain-adapted clones on days four (left) and ten (right) days post-inoculation. Cultures were inoculated with virus produced from the indicated clones. Culture media was replaced one day before collection at the indicated day and a 24-hour supernatant was then analyzed by ELISA to determine p27Gag levels.

Viruses derived from all six of the molecular clones replicated well in macrophages, and while the levels of p27Gag produced fluctuated between infections, the pattern of p27Gag production between the six viruses was remarkably consistent between experiments with the exception of the p27Gag production by clone 153, which induced strong p27Gag production on day 4 of this experiment, had reduced levels on day 10, and had inconsistent p27Gag production in subsequent experiments. Clones 109, 129, and 169 consistently produced the highest levels of p27Gag (Figure 1). Although strong p27Gag production was induced by clone153 on day 4 of this experiment, p27Gag levels were much reduced by day 10, and p27Gag production with this clone was inconsistent in subsequent experiments. As controls, the T-cell-tropic clones SIVmac239 and the molecular clone SIVmac251 were used and neither of these clones was able to produce detectable p27Gag after ten days, whereas the SIVmac251stock (the progenitor strain for SIVmac239, the SIVmac251 molecular clone and our microglia serial passage) successfully infected macrophages but produced relatively low levels of p27Gag (data not shown). Based on these results and the genetic similarity of the brain-adapted clones, subsequent experiments focused only on clones 129 and 169 as representatives of this particular genotype of SIV.

**Figure 2:**
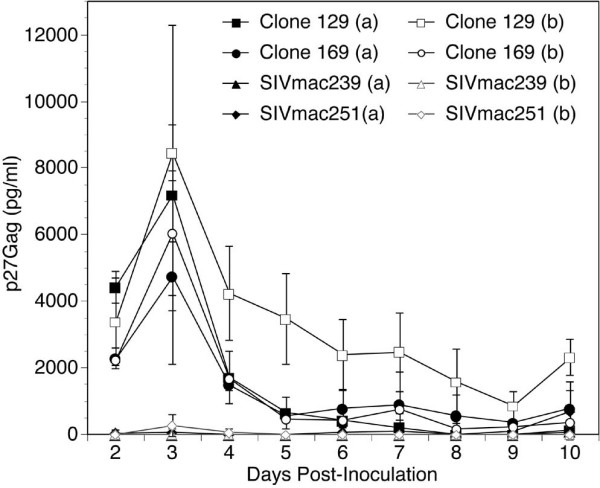
Daily SIV production in macrophage cultures. Macrophages from two different rhesus monkeys (a – 359, b – 420) were inoculated with virus produced from the indicated clones. Culture media was replaced each day and the removed supernatant was analyzed by ELISA to determine 24-hour p27Gag levels. This figure is representative of the infectious phenotype for these viruses in this cell type seen in four separate experiments.

Both the 129 and 169 molecular clones produced a similar infectious phenotype following spinoculation, producing p27Gag levels that peaked early after infection and then slowly declined (Figure 2). This particular infection phenotype, an early peak in p27 levels, was seen in all infections with either of these two viruses, although SIVmac129 consistently produced higher peak p27 levels than SIVmac169.

### Microglia Infection

Along with perivascular macrophages, microglia are the most commonly infected cells in the brain [31]. In order to determine if the molecularly cloned viruses were able to infect microglia, these cells were isolated from the brains of 3 animals (uninfected with SIV, but treated with methamphetamine for other studies). The microglia were spinoculated with virus prepared from clones129, 169, or SIVmac239. The molecular clone of SIVmac251 was also used to infect microglia from two of the three animals.

**Figure 3:**
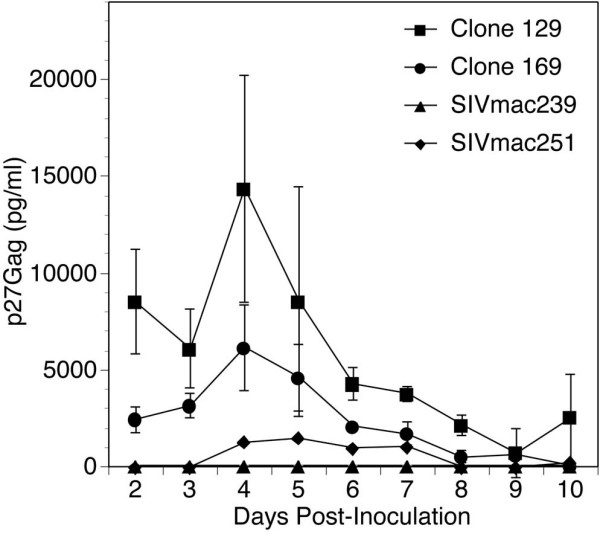
Daily SIV production in microglia cultures. Microglia were inoculated with virus produced from the indicated clones. Culture media was replaced each day and the removed supernatant was analyzed by ELISA to determine 24-hour p27Gag levels in the supernatant on each day of infection. This figure is representative of the infectious phenotype of these viruses in this cell type in three separate experiments using microglia from independent monkeys.

Viruses from both clones 129 and 169 were able to productively infect microglia, producing very high levels of p27Gag within the first 5 days, and then slowly declining out to day ten (Figure 3). While the peak levels of p27Gag production were reached more slowly in microglia than in macrophages, taking between 4 and 6 days rather than 3 or 4, the pattern of infection was similar to that seen in macrophage infections with these viruses. SIVmac239 was unable to infect microglia, failing to produce detectable levels of p27Gag in any of the infections. The SIVmac251 molecular clone was only able to infect microglia at a very low level, producing detectable p27Gag only sporadically during the course of infection (Figure 3).

### Spread in Macrophage Infection

**Figure 4:**
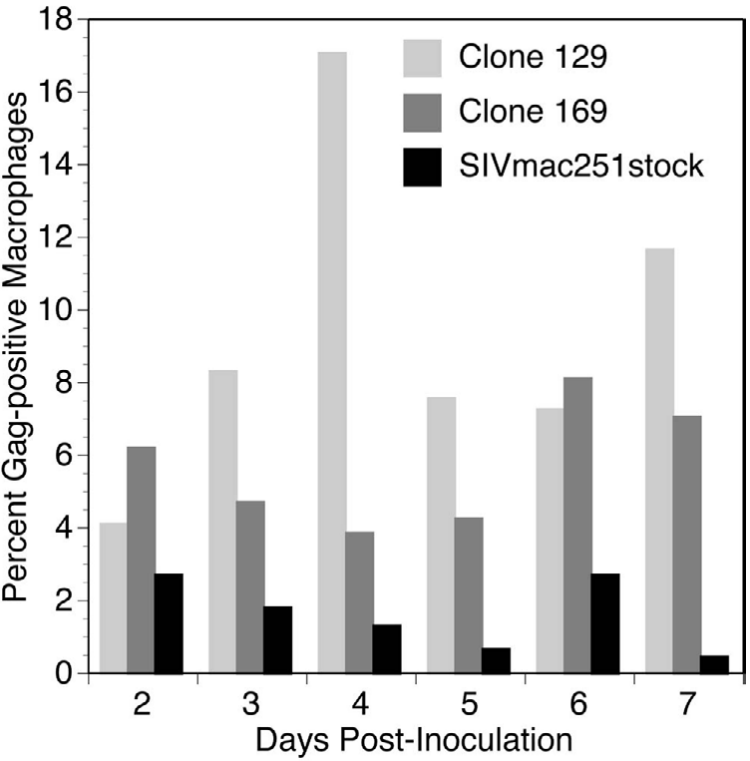
Infected cell percentage in macrophage culture. The percentage of macrophages infected each in chamber slide culture of primary macaque macrophages infected with brain-adapted clones 129 and 169 and the SIVmac251stock. In fourteen separate experiments, slides with primary macrophages from four different macaques were inoculated with SIV and then fixed and stained with DAPI and p27Gag at the indicated times. Data for each day are the average from these experiments.

Because macrophage tropism is a common characteristic of viruses found in the brains of individuals with neuroAIDS, the spread of virus between macrophages may carry important implications for understanding disease progression in the CNS. To assess spread of infection through macrophages, we enumerated the number of infected cells in cultures of primary macrophages inoculated with the viruses prepared from molecular clones 129 and 169, in comparison to the parental SIVmac251stock. In order to account for donor-related differences in macrophage infection, we examined infection of monocyte-derived macrophages in fourteen experiments, utilizing cells from four different macaques. The percentages of infected macrophages varied between experiments (most likely due to host-cell differences or the variability inherent in working with primary cells), but each viral clone produced infection within 48 hours of inoculation. Viruses generated from clones 129 and 169 both produced separate, unique infection patterns in all animals (Figure 4). In particular, clone 129 followed a similar infection pattern found by measuring supernatant p27Gag (shown in Figure 2), showing the highest percent of infected cells early on (17.1% by day 4). In contrast, clone 169 manifested the highest percent of infected cells later (8.2% on day 6) (Figure 4). The SIVmac251stock showed a very low level of infection, with 3% or less cells infected each day.

**Figure 5:**
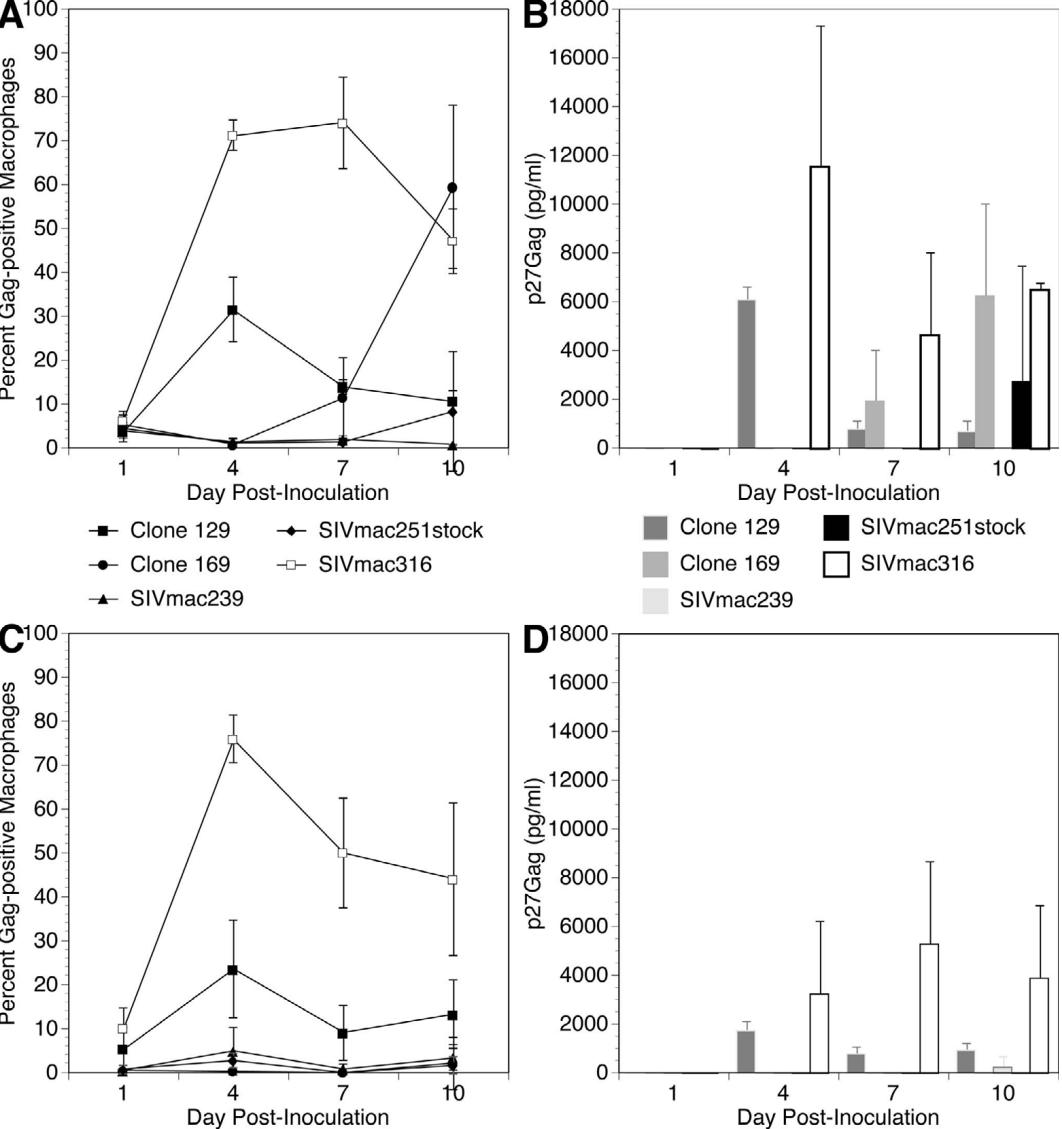
Spread and production of five different isolates of SIV in primary macrophages. Data from two monkeys (A, B – #408; C, D – #411) are shown for infected cell percentage (A, C) and supernatant p27Gag (B, D) from triplicate cultures in chamber slides.

To further examine the behavior of this brain-adapted phenotype in terms of viral spread, we performed a second macrophage infection over a 10-day time course, again using clones 129 and 169 as well as the SIVmac239 and SIVmac316 viruses, and the parental SIVmac251stock. Due to the limited number of macrophages derived from each animal, infections were only analyzed by staining and p27Gag analysis on days 1, 4, 7 and 10 post-infection. Since macrophages isolated from different rhesus macaques vary in their *in vitro *susceptibility to infection, in order to account for this variation, macrophages from macaques with different susceptibilities were used for each experiment. Although the percentages of infected cells (Figure 5A, 5C) and p27Gag levels (Figure 5B, 5D) varied between animals, the general infection pattern, with one notable exception, was remarkably similar.

Macrophages from donor monkey 408 showed no significant infection by any virus on day one post-inoculation, with all chambers showing a percentage (<3%) of infected cells and no detectable p27Gag levels in the supernatant (Figures 5A,B). Day four post-inoculation was much different, showing increases in percent of cells infected and p27Gag levels in chambers infected with SIVmac316 (71% of cells infected with p27Gag levels of 11.5 ng/ml) and clone 129 (30% and 6.1 ng/ml). Chambers inoculated with clone 169, SIVmac239 and SIVmac251stock had an extremely low infected cell percentage (<1%) and no detectable p27Gag levels in the supernatant. On day seven post-inoculation, the SIVmac316-infected cell percentage remained constant (66.6%) while p27Gag levels dropped (4.6 ng/ml). The percentage of infected cells in chambers infected with clone 129 dropped more than two-fold (12.8%) and supernatant p27Gag levels in the supernatant also dropped (0.8 ng/ml). No change was seen in SIVmac239 and SIVmac251stock infections. Surprisingly, the infected cell percentage in cultures inoculated with clone 169 greatly increased (14.6%), as did p27Gag levels in the supernatant (1.9 ng/ml). These trends all continued on day 10, with a greater than 3-fold increase in infected cell percentage (58.4%) and supernatant p27Gag levels (6.2 ng/ml). SIVmac316-infected chambers had reduced infected cell percentage (42.8%), though p27Gag levels in the supernatant increased (6.5 ng/ml). Chambers infected with clone 129 showed both reduced infected cell percentage (8.1%) and slightly reduced p27Gag levels in the supernatant (0.7 ng/ml). There was no change in the chambers infected with SIVmac239. Chambers inoculated with the SIVmac251stock showed increased infected cell percentage (5.5%) and a large increase in p27Gag levels in the supernatant (2.7 ng/ml) at this last time point.

Somewhat similar infection trends were seen on infection of macrophages from macaque 411 (Figure 5C,D). On day one post-inoculation, infected cell percentages in chambers inoculated with SIVmac316 (10.1%) and clone 129 (4.1%) were both higher than those seen in the 408 infections, but neither infection generated detectable p27Gag levels in the supernatant. Inoculation with SIVmac316 and clone 129 then followed the same general pattern found in the 408 infections above, with increases in infected cell percentage and p27Gag levels in the supernatant on day 4, followed by a decline on days 7 and 10. Supernatant p27Gag levels were in general lower than those found from the macrophages from macaque 408. However, in contrast to the results found in the other monkey's macrophages, here clone 169 did not lead to detectable infected cells or p27Gag levels in the supernatant at any point in the infection. Furthermore a low infected cell percentage was seen in SIVmac251stock and SIVmac239 infected chambers on days 4 and 10 respectively, but neither of these cultures had detectable p27Gag levels at any point during the infection.

## Discussion

To improve understanding of the viral factors that allow certain strains of HIV/SIV to induce brain disease, we analyzed molecular clones generated from SIVmac251stock through serial passage in infected microglia *in vivo*. After the final passage, several brain-adapted molecular clones were isolated from two macaques, 225 and 321, both of whom died with SIVE. Sequence analysis of the *env *and *nef *genes of these viral clones showed remarkable genotypic homology, as the all the brain-adapted clones differed from their consensus sequence by less than 0.55%.

Separate examination of the cytoplasmic tail of gp41 from uncloned viral sequence derived from the same microglia supernatant used to isolate the brain-adapted clones provided independent verification of these similarities. The uniqueness of this genotype is seen in comparison with other common SIVs like SIVmac239, SIVmac316 and SIVmac17E-Fr, as the brain-adapted genotype differs from the *env *and *nef *gene sequences of the other virus by three to five percent (Tables 3,4,5). Furthermore, uncloned viral sequence derived from both 225 and 321 microglia showed a large number of identical non-synonymous mutations in the gp41 cytoplasmic tail when compared with both SIVmac251 and SIVmac182.

Because of the way they were derived, the sequential differences from other SIVmac251 derived viruses and the exceptional homology between the separately isolated viral clones, the amino acid changes in these clones likely represent positive selection for adaptations beneficial towards survival and infection in the brain and CNS. The large number of identical non-synonymous mutations from the original SIVmac251 strain supports this idea, as non-synonymous mutations are only maintained if they are beneficial adaptations. When these brain-adapted clones are examined in light of their separate derivation from two different animals and the relative frequency of mutations during viral infection of macaques, the extraordinary homogeneity and uniqueness of the sequence of these brain-adapted clones, along with the identical and numerous non-synonymous mutations found in virus from both animals, strongly indicates that this genotype developed as a result of viral adaptation to the unique environment found in the CNS.

Numerous studies using SIV have linked brain infection to macrophage tropism [32-34], and indeed, virus from all of the brain-derived clones were macrophage tropic. Representative clones derived from each macaque (clone 129 from macaque 225, and clone 169 from macaque 321), were further characterized, and found not only to be infectious in primary macaque macrophages, but also in primary macaque CD4+ T-cells and primary macaque microglia. In addition to characterizing the tropism of the brain-adapted clones, the macrophage and microglia infection experiments also demonstrated a distinct, reproducible infectious phenotype associated with this viral genotype.

Numerous studies have analyzed macrophage-tropic viruses found in animals infected with the T-cell tropic clone SIVmac239, a phenomenon that is thought to be due to a series of amino acid changes in the envelope gene. Using site-directed mutagenesis, Mori and colleagues found five amino acid changes in the SIV envelope, V67M, K176E, G382R from the SU region and K573T, R751G from the TM region that increased p27Gag production in macrophage cultures [24]. Kodama and colleagues examined 10 viral clones derived from the brain of macaque 316-85, and found that all contained 9 amino acid changes in the envelope gene, including the V67M and G382R changes as well as seven additional changes in the SU region; T158A, D178N, P334L/R, D385N, V388A and P421S and R751G in the TM region [35]. As macrophage tropism is thought to be crucial to viral infection in the brain, the emergence of amino acid changes that contribute to this characteristic is not surprising in clones derived from the brain. Indeed, the brain-adapted clones from this study were found to contain numerous changes in Env, including the G382R and R751G changes mentioned above (see Table 3).

Macrophage tropism alone is not sufficient for induction of neurological illness [26], and many studies cite specific genes thought to be important in the induction of CNS disease. Mankowski and colleagues demonstrated the primacy of the envelope gene in neurovirulence in the development of SIV/17E-Fr, and examination of this clone by Flaherty and colleagues found macrophage tropism associated changes V67M, P334R and G382R in the envelope, along with several unique amino acid changes [23,26]. These and other studies demonstrate that while there is a group of amino acid changes associated with the macrophage tropic aspect of brain adaptation, it is an additional set of amino acid changes that allow a virus to successfully adapt to the environment of the brain. The brain-adapted clones described in this paper are a perfect example of this, with several macrophage tropism associated changes in the envelope, along with a group of entirely unique amino acid changes; seven in gp120 and seven in gp41.

However, other studies of brain adaptation in SIV find that specific Nef sequences are also important for infection and replication of virus in the brain, implying that similar neuroadaptive changes may also occur in the *nef *gene [26,36,37]. Barber and colleagues have noted five amino acid changes in Nef between SIVmac239 and SIV/17E-Fr, including two, P12S and E150K, that mediate distinct Nef/kinase associations and may be important in neuroadaptation [38]. Also a study of four pigtailed macaques infected with SHIV containing *nef *from an SIV background demonstrated that the majority of *nef *genes amplified from an animal with neurologic disease encoded two amino acid changes, T110A and A185T [39]. The brain-adapted genotype described in this paper does not contain any of the Nef changes seen in SIV/17E-Fr but it does contain the T110A residue, along with four other amino acid changes unique to this genotype among the viruses examined.

It is clear from the number of common changes found in various brain derived SIV clones that certain amino acid residues in Env and Nef are important to the adaptation of SIV to the CNS environment, including, but not limited to, those changes contributing to macrophage tropism. As with many derivatives of SIVmac251, the brain-adapted viruses described here do match the amino acids for several of the reversions noted in SIVmac239 described above, notably G382R and R751G in Env and T110A in Nef. The brain-adapted genotype described here also contains some amino acid differences from SIVmac251 and SIVmac239 that match other neurovirulent viruses like SIV17E/Fr, although it lacks the commonly seen truncation in the cytoplasmic region of gp41 and contains several amino acid changes that are unique to this group of viruses.

Unlike the macrophage-tropic and neurovirulent variants of SIVmac239 described above, the brain-adapted viruses isolated in this study were selected, or evolved from, a stock which could infect macrophages naturally in the course of infection, which was then preserved and selected for by subsequent passage through the brains of other animals. We had previously reported that analysis of brain proviral DNA for a portion of gp120 revealed selection of homogeneous sequences over the course of microglia passage [27]. Here, we have expanded these studies to the entire Env as well as Nef, examination of viral RNA, and characterization of infectious phenotypes in macrophage and microglia. Unlike many other studies with SIV, the changes found in the brain-adapted genotype described in this paper are an example of forward selection, rather than reversions that function largely in the context of the backbone of the non-macrophage-tropic non-brain-derived strain SIVmac239.

It is interesting to note that three of the amino acid changes found in gp41 of the brain-adapted clones are not found in the same region of their immediate progenitor, SIVmac182, therefore they developed during the course of infection in each animal. The presence of identical amino acid changes in viruses derived from two separate animals indicates that the genotype described by these clones results from convergent evolution rather than random mutation, and therefore the particular changes found in the genomes of these clones may be important to the natural adaptation of the virus to the brain.

It is worth noting that both clones 129 and 169 show a distinct, reproducible phenotype of infection characterized by an early peak in viral p27 production, usually in the first 2–4 days, followed by a gradual decline over the remainder of the experiment. While these clones have been shown to cause disease *in vivo*, the presence of this phenotype *in vivo *is still uncertain. However, if this phenotype does occur during *in vivo *infection, it could be a method by which brain-adapted SIV establishes residence in the brain, using an initial burst of virus to seed macrophages and microglia, which, once infected, lie low, allowing the neutralization sensitive macrophage-tropic virus to avoid immune detection until virus in the periphery has sufficiently weakened the immune system for successful virus replication in the brain. This approach might be particularly effective for this virus, given its ability to infect microglia and the low-turnover rate of that cell type, to establish a viral archive in the brain. However, this phenotype has only been shown to be distinct among the five viruses tested in these experiments and may also be an experimental artifact due to spinoculation or another aspect of our infection protocol, so the relevance of this particular phenotype to SIV remains uncertain.

Based on the data presented above; the method of isolation, the uniqueness of the Env and Nef sequences and their derivation through forward evolution in the brain and unique phenotype and tropism, it is clear that the genotype represented by the clones described in this study represents a new, brain-adapted genotype of SIV with a unique phenotype of infection. Further study of this genotype and its unique phenotype, both alone and in comparison with other brain-adapted strains of SIV, will hopefully generate greater understanding for the viral basis of brain disease and dementia, providing new targets and avenues of research in order to more effectively combat this disease.

## Methods & Materials

### Cell isolation and culture

All cell culture media and components were obtained from Invitrogen (Carlsbad, California), fetal calf serum (FCS) from Hyclone (Logan, Utah) and recombinant human macrophage colony-stimulating factor (M-CSF) from Peprotech (Rocky Hill, New Jersey). 174xCEM cells were obtained from the NIH AIDS and Reference Reagent Program (Germantown, Maryland), and grown in RPMI-1640 containing 10% FCS, 10 mM Hepes, and 100 U/mL penicillin, 100 μg/mL streptomycin and 250 ng/mL fungizone (added as an antibiotic cocktail called PSF).

Monocyte-derived macrophages were prepared from rhesus peripheral blood mononuclear cells (PBMC). PBMC were isolated by Histopaque 1077 (Sigma-Aldrich, St. Louis, Missouri) gradient centrifugation, and cultured at 2 × 10^6 ^cells/ml in complete macrophage media (RPMI-1640, 10% FCS, 5% autologous monkey serum, 10 mM HEPES, 10 ng/ml M-CSF, 1% PSF). Cultures were incubated briefly with PBS and then washed with serum-free RPMI-1640 on days 1, 3, and 5 post-isolation to remove non-adherent cells. After washing on day 5 post-isolation, cells were washed one additional time with PBS and then incubated in Versene (Invitrogen) on ice, agitating every 1–2 minutes. Cells were then shaken loose, enumerated in a Coulter Z2 counter (Beckmann-Coulter, Fullerton, California) and resuspended in complete macrophage media. Purity was ascertained by FACS analysis.

To prepare microglia, the meninges, surface vessels, and choroid plexi were removed from brains taken from sterile phosphate buffered saline-perfused rhesus monkeys. Then brain tissue was homogenized with a Tenbroeck tissue homogenizer, and microglia purified by collagenase/DNase digestion and Percoll gradient centrifugation as described [28]. Cells were enumerated in a Coulter Z2 counter, resuspended in complete microglia media (RPMI-1640, 10% FCS, 20 mM HEPES, 50 ng/ml M-CSF), and plated in 48-well plates at a concentration of 7.5 × 10^5 ^cells/well. Purity was ascertained by FACS analysis. Non-adherent cells are removed by washing 1, 3 and 5 days after isolation, and microglia used for experiments on day 6 post-isolation.

### Animal infection

Two rhesus macaques were infected intravenously with cell-free stock of SIVmac182, obtained by 3 generations of *in vivo *serial passage of SIVmac251 (44, 47). Both of these animals developed neuroAIDS with SIV encephalitis. Macaque 225 had a natural, rapid course of disease, requiring sacrifice at 80 days post-viral inoculation. Monkey 321 was treated with an anti-CD8 antibody at the time of infection [40] and also had a rapid course of disease, requiring sacrifice at 108 days post-viral inoculation. Following sacrifice, microglia from both animals were cultured as described above and cell-free supernatant was drawn from these cultures on days 8 through 12 post-isolation.

### Molecular cloning

Nucleic acids were isolated from the supernatants of cultured microglia, isolated from macaques 225 and 321, using a QIAamp Viral RNA mini kit (QIAGEN, Valencia, California) and were used to generate viral cDNA using the primer SIV GSP (see Table 1 for all primer sequences). Nested PCR were then used to amplify the cDNA, using the primers 10505 and 6516 for the first round of amplification, and primers 10505 and Sph2 for the second round of amplification. The PCR product was analyzed by gel electrophoresis to insure it was the proper size and then excised using the crystal violet gel excision system (Invitrogen). Approximately 10 ng of cDNA was cloned into the TOPO-XL vector (Invitrogen) and transformed into Max Efficiency STBL2 cells (Stratagene, La Jolla, California). Plasmid DNA was then isolated, restriction mapped, and initially sequenced using primer For8, corresponding to a region of the gp41 gene.

**Table 1:** Sequence of oligonucleotide primers used for reverse transcription, PCR, and sequencing of SIV.

**Primer**	**Sequence**
*Reverse Transcription*	
SIVGSP	TGCTAGGGATTTTTCCTGCYTCGGTTT
	
*Nested PCR*	
6516	CTCGCTTGCTAACTGCA CTTCTAATCATATCTA
Sph2	GCATGCTATAACACATGCTATTGTAAAAAGTGTT
10505	AAGCAGAAAGGGTCCTAACAGACCAGGGTCTTCA
	
*Molecular Clone Sequencing*	
For 1	AACTCAGTGCCTACCAGATAA
For 2	TGGCATGGTAGGGATAATAGGA
For 3	ATAAAAGAGGGGTCTTTGTGCT
For 4	AACTGCAGAACCTTGCTATCG
For 5	GTTTGATCCAACTCTAGCCTACAC
For 6	ATGACAGGGTTAAAAAGAGACAAGA
For 7	GAATTGGTTTCTAAATTGGGTAGA
For 8	GAGGCACAAATTCAACAAGAGAAG
For 9	CATACAGAAAACAAAATATGGATGA
For 10	TCCTGGTCCTGAGGTGTAATCCTG
Rev 1	CGCAAGAGTCTCTGTCGCAGAT
Rev 2	AGAGGGTGGGGAAGAGAACACTG
Rev 3	ACTTCTCGATGGCAGTGACC
Rev 4	CCAGACATAATGGAGACTGGTAA
Rev 5	AGAGTACCAAGTTTCATTGTACTC
Rev 6	AGGCAAATAAACATTTTTGCCTAC
Rev 7	GAGCGAAATGCAGTGATATTTATACATCAAG
	
*Population PCR and Sequencing*	
8877For	ATAGCTGGGATGTGTTTGGC
8534For	GCTGGGATAGTGCAGCAACAGCAAC
8406For	CTACTGGTGGCACCTCAAG
9452Rev	CGAGTATCCATCTTCCAC
9625Rev	CCTACCAAGTCATCATCTTCCTCA
9880Rev	ATCCTCCTGTGCCTCATCTG
10203Rev	ATCAAGAAAGTGGGCGTTCCCGACC

### 174xCEM Transfection and Infections

Plasmid DNA was prepared in STBL2 cells (Invitrogen) and then isolated by miniprep (QIAgen). Clones were prepared for ligation by digestion with SphI, phenol extraction and ethanol precipitation. Following this preparation, 0.4 μg of the plasmid containing the newly-derived 3'-regions of the viral cDNA was ligated to 1.6 μg of an SphI/SalI restriction digest of the previously characterized 5' fragment of the SIV genome, constituting the first 6516 bp of SIVmac239 (p239SpSp5, NIH AIDS Research and Reference Reagent Program), which had been subcloned into the Litmus38 vector (New England BioLabs, Beverly, Massachusetts). This process was also used to combine the 3' regions of SIVmac239, and the molecular clone SIVmac251, both originally obtained from the NIH AIDS Research and Reference Reagent Program, with the 5' region of SIVmac239.

The ligation products were transfected into 174xCEM cells by DEAE-Dextran. Transfections were observed for syncytia for 18 days and supernatant was taken from each culture periodically for use in p27Gag ELISA (SIV Core Antigen Assay, Beckmann-Coulter) to test for the presence of infectious virus. Clones were monitored microscopically for the speed of syncytia formation and speed of cell death in culture, and the media was assayed by ELISA for p27Gag level in order to characterize the robustness of infection. All transfections were each performed independently at least twice, with an extra transfection being performed on clones with inconsistent results in the first two transfections.

Viral stocks were produced in 174xCEM cells using either transfection, as above, or infection, for the uncloned SIVmac251 stock and a viral stock of the SIVmac239/316EM*/nef-open molecular clone. SIVmac239/316/Macropahge Variant is a macrophage tropic derivative of SIVmac239 (this clone is hereafter referred to as SIVmac316, kindly provided by Dr. R. Desrosiers, New England Primate Research Center, Southborough, Massachusetts). Following infection or transfection, when syncytia become prevalent throughout the infected culture, cells were washed and resuspended in fresh growth media every twenty-four hours, with the cell-free supernatant aliquoted and stored at -80°C. Each stock was run in triplicate p27Gag ELISA for quantification.

### Sequence Analysis

The 3' regions of nineteen molecular clones were completely and bi-directionally sequenced using a battery of primers designed to cover the full length of that fragment of the genome. The group of primers used included M13 Forward and M13 Reverse as well as ten SIV-specific forward primers and seven SIV-specific reverse primers, listed on Table 1. All sequencing reactions on the molecular clones were performed at the TSRI Nucleic Acids Core Facility.

Additional sequencing reactions were performed on uncloned cDNA prepared from cell-free viral RNA isolated from SIVmac251 stock, SIVmac182, and from cultured microglia from macaques 225 and 321. RNA was converted to cDNA using the First Strand cDNA synthesis kit (Marligen Biosciences, Ijamsville, MD) and amplified by using the primers 8534For, 8406For, 9880Rev and 10203Rev, followed by purification of the PCR product and sequence analysis with 8777For, 9452Rev and 9625Rev in order to sequence the gp41 cytoplasmic tail region (bases 8750–9499). All direct sequencing reactions of PCR products were performed by Retrogen (San Diego, California).

### Macrophage and Microglia Infection

Macrophages were purified as above, and plated in 48-well plates at 7.5 × 10^4 ^per well. The macrophages were grown in these plates for one day and then inoculated in triplicate with 4 ng (p27Gag) of the virus stock in complete macrophage media. Infection was facilitated by spinoculation (30). Briefly, virus was added to each well and plates were centrifuged at 1,200 × g for 2 hours at 25°C. Plates were then incubated at 37°C for 22 hours, washed twice with Hanks Balanced Salt Solution (Invitrogen), and fed with complete media. Every 24 hours, supernatant was removed and stored at -80°C, while cells were fed with fresh complete macrophage media.

Microglia were infected following the above spinoculation protocol. Following spinoculation media was collected and replaced with fresh complete microglia media daily, similar to the collection protocol followed in macrophage infections.

### Macrophage chamber slide culture infection and analysis

PBMCs were isolated and enumerated as described above, and monocytes purified using the MiniMacs magnetic separation system (Miltenyi Biotech, Cologne, Germany) using paramagnetic anti-CD11b beads. The cells were then enumerated and resuspended in complete macrophage media and plated onto 8-well chamber slides (Fisher Scientific, Pittsburgh, Pennsylvania) at 10^5 ^cells per well.

Media was changed on day 3 post-isolation, and on day 6 removed and replaced with the viral inoculum containing 6 ng (p27Gag) of virus stock diluted in complete macrophage media, performed in triplicate. Slides were incubated for 24 hours at 37°C, after which the supernatant was gently aspirated, cells washed, and fed with complete macrophage media. Day one post-inoculation and every three days following, one set of slides (corresponding to 3 wells for each infecting virus and one uninfected well for each virus) was processed for staining and the supernatant from each chamber was carefully aspirated and stored at -80°C.

Each chamber was then washed once with phosphate buffered saline (PBS, Invitrogen) and fixed by incubating 20 minutes at room temperature in 3% paraformaldehyde/PBS. After fixing, chambers were gently washed with distilled water and incubated in 5% bovine serum albumin (BSA) in PBS for five minutes. Cells were incubated for 1 hour with a 1:100 dilution of FA2 (mouse anti-p27Gag antibody, originally obtained from the NIH AIDS Research and Reference Reagent Program) in 5% BSA in PBS. Following this incubation, chambers were washed once with 1% BSA in PBS, and incubated for 1 hour with a 1:500 dilution of rhodamine-conjugated goat-anti-mouse antibody (Molecular Probes, Eugene, Oregon) in 5% BSA in PBS. Cells were then washed once with PBS and incubated for 3 minutes with 4,6-diamidino-2-phenylindole dihydrochloride hydrate (DAPI, Sigma-Aldrich) diluted to 10 ug/mL in PBS. All of these reactions were performed at room temperature in the dark. Cells were then gently washed once with PBS, chamber divisions were removed and coverslips were attached using Vectashield mounting medium (Vector Labs, Burlingame, California) and sealed with nail polish. Slides were stored in the dark at 4°C.

One day after staining, each set of slides was assessed by computer-assisted fluorescence microscopy using the Axiovision program (Carl Zeiss, AG, Germany). Three 10x fields were assessed from each chamber using rhodamine and DAPI filters, generating nine fields per virus per day examined. Three fields taken of an uninfected control well were used to control for background fluorescence. All observation and focusing was performed under DAPI fluorescence to avoid experimenter bias. The images were analyzed using OpenLab (Improvision, Lexington, MA) to elucidate the total number of cells and infected cells in each field. The total number of cells per field was determined by counting the DAPI-stained nuclei, while the number of infected cells in each field was determined using Boolean operations to overlay the FA2-Rhodamine stained viral gag with those nuclei. The number of positive-staining cells in the control wells was used to account for background fluorescence.

## Competing interests

The author(s) declare that they have no competing interests.

**Table 6:** Predicted amino acid residue at the indicated location in Nef. Bold indicates unique amino acids in clones 129 and 169.

**Nef**	**7**	**12**	**39**	**43**	**49**	**53**	**75**	**93**	**110**	**112**	**119**	**150**	**201**	**206**
SIVmac239	M	P	Y	P	G	R	E	E	T	S	M	E	S	P
SIV/17E-Fr	M	S	Y	P	G	L	K	Q	T	S	I	K	S	P
SIVmac251	R	P	S	L	G	L	E	E	T	S	M	E	S	P
SIVmac32H	R	P	S	L	G	L	E	E	T	S	M	E	S	P
SIVmac1A11	M	P	S	L	G	L	E	E	T	S	M	E	S	P
Clone 129	R	P	S	L	**D**	R	E	E	**A**	**T**	M	E	**A**	**S**
Clone 169	R	P	S	L	**D**	R	E	E	**A**	**T**	M	E	**A**	**S**

**Table 7:** Predicted amino acid residues in the gp41 cytoplasmic tail that differ in the indicated viruses. Residues found only in the microglia-passage derived SIVmac182 stock, and/or in the resulting virus released from monkey 225 and 321 microglia, are in bold; italics further indicated residues found in the microglia virus but not SIVmac182. For amino acid 747, a mixed sequence was present in SIVmac182 capable of encoding either amino acid listed. Population-based sequencing was performed on the SIVmac251, SIVmac182, 225 microglia, and 321 microglia viral stocks. SIVmac239 is provided for reference.

	**695**	**737**	**741**	**747**	**748**	**751**	**758**	**760**	**785**	**794**	**802**	**808**	**821**	**831**	**837**	**850**	**858**
SIVmac239 (clone)	V	I	P	E	S	R	G	S	S	V	L	T	T	H	V	G	R
SIVmac251 (stock)	V	I	P	E	S	G	S	S	S	A	L	A	T	Q	G	G	G
SIVmac182 (stock)	**I**	I	**Q**	**G/E**	**G**	G	G	**R**	S	V	**F**	T	T	Q	G	**R**	R
225 microglia	**I**	**T**	**Q**	**G**	**G**	G	G	**R**	**N**	V	**F**	T	**A**	Q	G	**R**	R
321 microglia	**I**	**T**	**Q**	**G**	**G**	G	G	**R**	**N**	V	**F**	T	**A**	Q	G	**R**	R
